# Assessment of Endothelial Dysfunction in Childhood Obesity and Clinical Use

**DOI:** 10.1155/2013/174782

**Published:** 2013-04-03

**Authors:** Luc Bruyndonckx, Vicky Y. Hoymans, Amaryllis H. Van Craenenbroeck, Dirk K. Vissers, Christiaan J. Vrints, José Ramet, Viviane M. Conraads

**Affiliations:** ^1^Laboratory of Cellular and Molecular Cardiology, University Hospital Antwerp, Wilrijkstraat 10, 2650 Edegem, Belgium; ^2^Cardiovascular Diseases, Department of Translational Pathophysiological Research, Faculty of Medicine and Health Sciences, University of Antwerp, Universiteitsplein 1, 2610 Antwerp, Belgium; ^3^Department of Pediatrics, University Hospital Antwerp, Wilrijkstraat 10, 2650 Edegem, Belgium; ^4^Department of Nephrology, University Hospital Antwerp, Wilrijkstraat 10, 2650 Edegem, Belgium; ^5^Faculty of Medicine and Health Sciences, University of Antwerp, Campus Drie Eiken, Universiteitsplein 1, 2610 Antwerp, Belgium; ^6^Department of Cardiology, University Hospital Antwerp, Wilrijkstraat 10, 2650 Edegem, Belgium

## Abstract

The association of obesity with noncommunicable diseases, such as cardiovascular complications and diabetes, is considered a major threat to the management of health care worldwide. Epidemiological findings show that childhood obesity is rapidly rising in Western society, as well as in developing countries. This pandemic is not without consequences and can affect the risk of future cardiovascular disease in these children. Childhood obesity is associated with endothelial dysfunction, the first yet still reversible step towards atherosclerosis. Advanced research techniques have added further insight on how childhood obesity and associated comorbidities lead to endothelial dysfunction. Techniques used to measure endothelial function were further brought to perfection, and novel biomarkers, including endothelial progenitor cells, were discovered. The aim of this paper is to provide a critical overview on both *in vivo* as well as *in vitro* markers for endothelial integrity. Additionally, an in-depth description of the mechanisms that disrupt the delicate balance between endothelial damage and repair will be given. Finally, the effects of lifestyle interventions and pharmacotherapy on endothelial dysfunction will be reviewed.

## 1. Introduction

Society is faced with a new pandemic. Obesity and its associated non-communicable diseases, such as cardiovascular (CV) complications, diabetes [1], sleep apnea [2], and asthma, are a major threat to the management of health care worldwide. It may seem paradoxical but both childhood malnutrition in developing countries and the rapidly increasing prevalence of overweight and obesity in Western youth have a common denominator, low income [3].

Being obese as a child comes at a price. Childhood overweight and obesity increase the risk of obesity at adult age [4] and are associated with CV risk factors. A high BMI during childhood and adolescence has been associated with, respectively, premature death from disease and increased risk of coronary heart disease at adult age [5, 6]. Interestingly, however, in a recent meta-analysis of four studies, overweight and obese children who became nonobese in adult life were not different in terms of several risk parameters of cardiovascular disease to those patients who had never been obese [7]. This could be explained by the fact that other indicators of a healthy lifestyle, such a regular physical exercise, were not taken into account. During the past two decades, the concept of early vascular changes, which act as the primum movens for future CV complications, has been tested [8]. The development and refinement of technical tools that allow the *in vivo* evaluation of endothelial dysfunction, which is considered the earliest demonstrable feature of atherosclerosis, rapidly advanced pathophysiological insights. Recently, more fundamental research into disrupted bone-marrow-related endothelial repair mechanisms (i.e., endothelial progenitor cells), as well as the identification of novel markers of endothelial damage (i.e., endothelial microparticles), has created an entirely new line of research [9, 10]. These biomarkers hold promise in terms of designing effective strategies and to evaluate their effect in the combat against the devastating consequences of childhood obesity.

A long-lasting change of unhealthy lifestyles is fundamental in helping obese children and their parents to fight this disease. Therefore, a multidisciplinary approach to adopt physical activity and balanced calorie restriction into daily life is inevitable to counterbalance the attraction of highly processed food, motorized transportation, and TV-related sedentarism.

This paper will provide a critical overview on both *in vivo* as well as *in vitro* markers for endothelial integrity. As an introduction we will first describe the obesity-induced imbalance of endothelial repair and damage, resulting in early endothelial dysfunction. Currently available data on the effect of lifestyle changes as well as possible pharmacological interventions to counteract obesity-induced endothelial malfunction will be recapitulated. Although the focus of this review is on childhood obesity, it is important to stress that data from obese adults are preponderant and therefore will be incorporated in the text. Comparison of the available literature will expose differences and stresses the fact that the scientific community should invest more in childhood obesity research since, indeed, children are not small adults. In addition, the value of longitudinal studies, starting early in childhood, cannot be underestimated when it comes to exactly decipher the timing of pathophysiological events.

## 2. Endothelial Dysfunction

**Figure 1:**
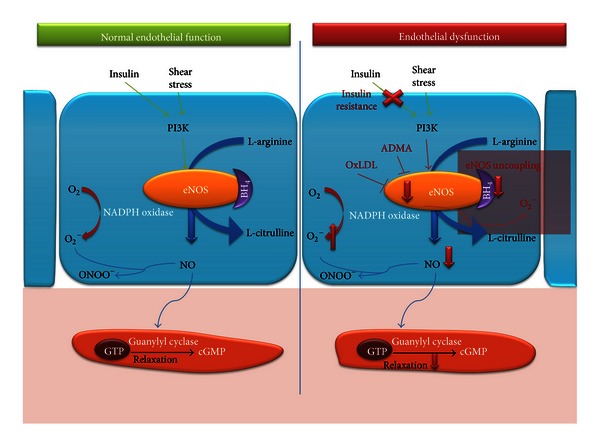
Normal endothelial function versus endothelial dysfunction. Schematic overview of nitric oxide (NO) production and relaxation of Vascular Smooth Muscle Cells (VSMC). In response to increased shear stress or as a result of insulin signaling, the phosphoinositol 3 kinase (PI3K)/akt pathway is activated leading to phosphorylation of endothelial Nitric Oxide Synthase (eNOS). eNOS, together with the necessary cofactor tetrahydrobiopterin (BH_4_), converts L-arginine to L-citrulline and NO. NO activates guanylyl cyclase, which induces smooth muscle relaxation, through increased production of cyclic Guanosine MonoPhosphate (cGMP). Superoxide reduced NO bioavailability by reacting with NO to form peroxynitrite (ONOO^−^), which has strong oxidant properties. Endothelial dysfunction in obese children is characterized by insulin resistance impairing insulin-mediated NO production and subsequent vasodilation. Furthermore, oxidized LDL and ADMA are inhibitors of eNOS activation. In the situation of diminished availability of BH_4_, eNOS becomes “uncoupled” and paradoxically leads to increased reactive oxygen species (ROS) generation, which also contributes to reduced bioavailability of NO and vasoconstriction. ADMA, Asymmetric DiMethylArginine; PI3K, PhosphatidylInositol 3-Kinase; BH_4_, tetrahydrobiopterin; eNOS, endothelial Nitric Oxide Synthase; O_2_^−^, superoxide; ONOO^−^, peroxynitrite; GTP, Guanosine TriPhosphate; cGMP, cyclic Guanylyl MonoPhosphate; NADPH oxidase, Nicotinamide Adenine Dinucleotide Phosphate oxidase; OxLDL, Oxidized Low-Density Lipoprotein Cholesterol.

The endothelial cell-layer is located at the border between circulating blood and vascular smooth muscle cells (VSMC) [11]. In response to stimuli that indicate increased blood flow demand (e.g., increased shear stress), activation of the PhosphoInositol 3-Kinase (PI3K)/Akt pathway will lead to phosphorylation of endothelial Nitric Oxide Synthase (eNOS), which is necessary for its activation and the generation of Nitric Oxide (NO) (Figure 1) [12]. By fine-tuning vascular smooth muscle relaxation, NO is one of the main regulators of vascular tone [13, 14]. In addition, healthy endothelium is responsible for the maintenance of an atheroprotective environment: it prevents platelet aggregation, proliferation of VSMC, and adhesion and subsequent diapedesis of leukocytes through the vascular wall [15].

The common and straightforward definition of endothelial dysfunction is “an imbalance between vasodilating and vasoconstricting substances produced by (or acting on) endothelial cells” [16]. The close interaction between endothelial cells and their environment, however, highlights the process complexity. Indeed, due to its location within the vascular wall, the endothelium is directly exposed to damaging factors such as high blood pressure and elevated lipid levels, which are common in obese subjects. The notion that endothelial dysfunction is a necessary and first step towards atherosclerosis has led to a tremendous interest and research investment in an attempt to unravel underlying pathophysiological mechanisms [17].

Fortunately, endothelial dysfunction is not an irreversible process. Upon endothelial damage and ischemia, repair mechanisms are activated. The concept of endothelial homeostasis, which is the net result of endothelial damage and repair, has provided the basis for developing novel therapeutic options, as well as a fascinating new research domain of endothelial “rejuvenation.”

### 2.1. Factors Contributing to Endothelial Dysfunction in Obesity

Similar to adults, CV risk factors tend to cluster in obese children and include hypertension, high cholesterol and triglycerides, insulin resistance, a proinflammatory status, disturbances in adipocytokines, and physical inactivity [18]. As a result, assessing the specific contribution of one sole risk factor is extremely difficult. In the following, we will briefly cover recent insights into factors causing endothelial dysfunction in childhood obesity.

#### 2.1.1. Hypertension

Hypertension is prevalent in obese children. Based on data gathered in the Avon Longitudinal Study of Parents and Children (ALSPAC; 7589 children aged 8.8–11.7 years), the odds ratio for hypertension was 10.7 (95% CI 7.2–15.9) for obese boys and 13.5 (95% CI 9.4–19.5) for obese girls [18], compared to children with normal weight. The interaction between endothelial dysfunction and hypertension is complex but recent data from longitudinal population studies do not support a bidirectional relationship.

In a cross-sectional and longitudinal study of 3500 adults, Shimbo et al. showed that hypertension is more prevalent in patients with low flow-mediated dilation (FMD) [19]. However, reverse findings could not be confirmed since impaired endothelial function was not predictive of incident hypertension. Similar findings were seen in The Cardiovascular Risk in Young Finns Study, where elevated systolic blood pressure in adolescent boys predicted impaired brachial endothelial function 21 years later in adulthood [20]. A recent analysis of data derived from the ALSPAC study [21] sheds light on the vascular consequences of obesity with time. In their study of 6,576 prepubertal children (aged 10 to 11 years; 80% normal weight, 16% overweight and 4% obese), obesity was associated with higher blood pressure, higher heart rate, and higher resting and hyperemic blood flow, all of which are compatible with a higher cardiac output state. Counterintuitively, these obese children had larger baseline brachial artery diameters, higher FMD, and lower arterial stiffness, despite an unfavorable metabolic profile. Although speculative, the authors propose an initial adaptive response, which ultimately, with longer exposure to obesity, will fail and culminate in vascular damage. Data derived from the Cardiovascular Risk in Young Finns Study support this view and demonstrate an adverse impact of CV risk factors including adiposity, high LDL cholesterol and high insulin level on the progression of intima-media thickness (IMT) in young adults aged 30 years [22]. Previous results from this longitudinal study provide further evidence for this hypothesis, since a correlation was found between CV risk factors and IMT only for participants with demonstrated endothelial dysfunction [23]. 

Obesity increases blood pressure through multiple mechanisms, which also affect endothelial function. These factors include increased activity of the renin-angiotensin aldosterone system (RAAS), as well as sympatho-activation. Angiotensin II, one of the main hormones in the RAAS system, also directly impairs NO production by affecting eNOS activity [30]. Increased sympathetic nervous activity leads to peripheral vasoconstriction and impairment of endothelial function [31]. Importantly, hypertension reduces bioavailability of NO and increases oxidative stress, due to increased Reactive Oxygen Species (ROS) generation and lower antioxidant capacity. In addition, Asymmetric DiMethyl Arginine (ADMA), a natural inhibitor of eNOS, has been found in higher concentrations in obese individuals [32].

#### 2.1.2. Lipids

High levels of low-density lipoprotein (LDL) cholesterol and low levels of High-Density Lipoprotein (HDL)-cholesterol are well-known independent CV risk factors. Native LDL can impair endothelial function by decreasing NO bioavailability and eNOS activity. However, the effect is more pronounced when LDL is taken up by macrophages and oxidized to oxidized LDL [33]. HDL cholesterol reduces vascular tone by increasing NO bioavailability; it reduces the expression of adhesion molecules for leukocytes and increases endothelial integrity by upregulating endothelial cell migration and proliferation [34, 35].

Results from the PEP Family Heart Study in 3038 adolescents (12 to 18 years) have demonstrated that central obesity, defined as elevated waist circumference and/or waist-to-hip ratio, is an independent predictor of hypertension, fasting glucose, elevated triglycerides, LDL-cholesterol, non-HDL-cholesterol, triglyceride/HDL-cholesterol ratio, low HDL-cholesterol, and risk factor clustering [36]. Yet, despite having central obesity, both prevalence of an unfavorable lipid profile as well as absolute concentrations of lipids were less pronounced than those found in obese adults. For instance, the prevalence of increased triglyceride concentration was 5,8% and 11,2% in obese male and female children, respectively, but reached approximately 25% in a large cohort of Italian obese adults [37].

Recent research supports the hypothesis that the well-known beneficial effects of HDL, such as macrophage cholesterol efflux and endothelial NO stimulation, are highly heterogeneous and may be altered in patients with coronary artery disease as well as diabetes. Therefore, HDL dysfunction has to be taken into account when novel HDL-targeted therapeutic interventions, such as Cholesteryl Ester Transfer Protein (CETP) inhibitors, are introduced [38].

#### 2.1.3. Physical (In)Activity

Physical activity is associated with a significant reduction in CV mortality in adult men and women [39–41], whereas physical inactivity predicts the development of overweight and obesity [42].

Using a questionnaire, physical activity in more than 6000 children between 11 and 19 years was investigated by De Bourdeaudhuij et al. Overweight children spent on average 5.27 ± 4.65 hours per week on physical activity versus 6.21 ± 5.07 hours per week for normal weight children. Significant differences were noted for vigorous and moderate physical activity as well, yet no differences in sedentary behavior were noted [43].

Similar to adults, endothelial function relates to fitness and physical activity in children, yet correlations are affected by age. In young children (5–10 years: mean age 8 years), physical activity was the strongest predictor of FMD in multivariate analysis [44]. Slightly older children (*n* = 145 (59 male, 86 female) with a mean age of 10.3 ± 0.03 years) demonstrated a significant correlation between FMD and the time spent at moderate and high intensity physical activity [45]. Correlation in that study with physical activity was strongest in the lowest tertile of endothelial function, suggesting that these children could benefit most from physical training. Endothelial function was also clearly related to fitness, objectively expressed as peak oxygen consumption during cardiopulmonary exercise testing. Based on a large study, involving 483 13-year-old adolescents, multivariate analysis pointed out that endothelial function correlated significantly to leisure time physical activity. This finding was only confirmed in boys, leading the authors to hypothesize that the overall lower physical activity seen in girls may explain these results [46]. A longitudinal study in children further demonstrated that an extensive increase in physical activity led to a significant improvement of FMD as well as less progression of intima-media thickness [47].

#### 2.1.4. Adipokines

Normal adipose tissue consists of adipocytes, and furthermore macrophages, fibroblasts, endothelial cells, and preadipocytes are present in the so-called vascular-stromal fraction [48]. Although it has long been thought that storage and release of free fatty acids were its only functions, adipose tissue is currently seen as an important endocrine organ, regulating glucose and lipid metabolism and producing a vast amount of cytokines (adipocyte-derived cytokines or adipokines) and hormones [49, 50]. Obesity is characterized by hypertrophic adipose tissue. As an effect of hypercaloric intake, adipocytes enlarge and release more free fatty acids, which activate macrophages to produce Tumor Necrosis Factor-alpha (TNF-*α*). As a consequence, adipocyte expression of Intercellular Adhesion Molecule-1 (ICAM-1), InterLeukin (IL)-6, and Macrophage Chemo attractant Protein-1 (MCP-1) is enhanced, promoting diapedesis of monocytes from the circulation to adipose tissue. With the exception of adiponectin, adipocytokine levels rise with obesity. Growing adipose tissue poses higher demands in terms of oxygen and nutrient delivery. Despite vasculogenesis [51], local hypoxia induces several angiogenic factors, which further suppress adiponectin expression even more and upregulate leptin production. This “hypersecretion of proatherogenic, pro-inflammatory, and prodiabetic adipocytokines which is accompanied by a decreased production of adiponectin” is called adipose tissue dysfunction [52]. Of the adipokines, leptin and adiponectin have a proven direct influence on angiogenesis and the endothelium, and are therefore further discussed [53].

*Leptin.* Leptin is a cytokine, which is mainly synthesized by white adipose tissue and released into the circulation. Its physiological role is to suppress appetite and to increase energy expenditure via the hypothalamus. However, circulating levels of leptin contradictorily rise with increasing body fat percentage, while obese patients have no diminished appetite [54].

Leptin has several proangiogenic effects, including enhanced Akt-mediated eNOS phosphorylation [55] as well as stimulation of endothelial cell proliferation [56], which are expected to be beneficial in the setting of endothelial dysfunction in obese patients. However, leptin also exerts proatherogenic effects, including the induction of ROS, a pro-inflammatory vascular effect, and stimulation of the proliferative capacity of VSMC [57].

These insights have led to the concept of a selective leptin resistance for both central (appetite) and peripheral pro-angiogenic effects, without any changes in terms of pro-atherogenic stimuli. The inverse correlation between leptin and endothelial function, independent of the metabolic and inflammatory disturbances associated with obesity adds, further supports to this hypothesis [58].

*Adiponectin.* Adiponectin is a 30 kDa protein produced by adipocytes with anti-inflammatory, antiatherogenic, and insulin sensitizing effects. This adipokine plays a central role in lipid and energy metabolism. Contrary to all other adipokines, adiponectin concentrations are lower in obese. In their elegant paper, Torigoe et al. demonstrated that even in healthy young men, circulating concentrations of adiponectin determine endothelial function [59], through eNOS mRNA stabilization and eNOS phosphorylation [60]. Moreover, high-glucose level-induced generation of ROS by endothelial cells is suppressed by adiponectin [61]. Recent human data show that adiponectin stimulates the migratory capacity of circulating angiogenic cells [62], thereby supporting animal experiments that suggested angiogenesis-stimulating effects [63] and the promotion of neovascularization [64].

#### 2.1.5. Inflammation

Obesity, and visceral obesity in particular, is associated with a low-grade pro-inflammatory status [65]. Upon activation, endothelial cells express adhesion molecules, which allow leukocytes to adhere and initiate a cascade of inflammatory reactions. Inflammatory cytokines produced by macrophages in adipose tissue (IL-18, TNF-*α*) further add to the expression of adhesion molecules. Not surprisingly inflammatory cytokines (e.g., C-Reactive Protein or CRP) are also associated with CV risk [66], and their concentration inversely correlates with endothelial function [67]. Another important mediator is IL-6 [68], which not only is a potent stimulator for the production of CRP, but also stimulates angiotensin II in VSMC and the associated production of ROS [69]. Besides reacting with NO, and thereby neutralizing its vasodilatory effect, ROSs also reduce eNOS activity and consequently NO production. Moreover, ROSs are able to directly inhibit eNOS and react with tetrahydrobiopterin (BH_4_), a necessary cofactor for eNOS activity [70]. In this situation, eNOS becomes “uncoupled” and paradoxically leads to increased ROS generation [71].

#### 2.1.6. Insulin Resistance and Type 2 Diabetes Mellitus

Under physiological conditions, insulin is a potent vasodilator, via the stimulation of the PI3K/Akt pathway to augment NO production. However, in the setting of insulin resistance this reaction is absent [72]. Yet insulin is still able to activate the Extracellular signaling-Regulated Kinase (ERK1/2) pathway leading to production of EndoThelin 1 (ET-1) and thus vasoconstriction [73]. The same pathway also leads to expression of adhesion molecules like Vascular Cell Adhesion Molecule (VCAM)-1 on endothelial cells [74]. Endothelial dysfunction per se can even contribute to insulin resistance, since impaired microvascular vasodilation in skeletal muscle reduces delivery of insulin and glucose to skeletal muscle and thereby causes insulin resistance [75].

In type 2 diabetes mellitus, NO bioavailability is significantly diminished: a reduction in eNOS activity [76] as well as eNOS uncoupling [77] and increased generation of ROS have been demonstrated [78] and further deteriorate endothelial function. Hyperglycemia in diabetic patients leads to production of Advanced Glycation End products (AGE) after reacting with proteins, lipids, and nucleic acids. AGE can induce the expression of ROS via NF-kappa B activation and TNF-*α* [79]. Type 2 diabetes is additionally characterized by increased levels of ET-1 [80]. Interestingly, a recent double-blind-placebo controlled trial in 46 type II diabetes patients with microalbuminuria demonstrated that 4 weeks of treatment with an oral ET-1 receptor antagonist (bosentan) can repair endothelial function [81].

## 3. Clinical Assessment of Endothelial Dysfunction

The landmark study by Ludmer et al. [82] led to the concept of endothelial dysfunction as the primum movens of the atherosclerotic process. The consequences of an abnormal vasodilator response (i.e., impaired vasodilation and even paradoxical vasoconstriction of coronary arteries upon the administration of acetylcholine) have thereafter been extensively studied. Epicardial and microvascular coronary endothelial dysfunction predicts CV events in patients with and without coronary artery disease [83]. Obviously, acetylcholine infusion can be applied during coronary angiography, but the invasive character prohibits its use in healthy individuals and children. Since then, novel, less invasive techniques have been developed, which will be discussed in detail in the following paragraphs.

### 3.1. Flow-Mediated Dilation

In 1992, Celermajer et al. described a noninvasive technique that allows assessment of peripheral endothelial function [84]. Measurement of flow-mediated dilation (FMD) at the level of a large conduit artery, usually the brachial artery, has since then become the most applied technique [85]. Briefly, high-resolution ultrasound is used to measure the internal diameter of the brachial artery, from lumen-intima interface on the near and far vascular wall [86]. After assessing baseline diameter, the brachial artery is occluded during 5 minutes using a sphygmomanometer inflated to suprasystolic pressure. When the cuff is deflated; the increased flow causes endothelial-dependent dilation through raised shear stress [84]. It is this proportional response (i.e., related to baseline diameter) that is significantly impaired in the case of endothelial dysfunction. 

Pathophysiological research confirms that this effect is mainly mediated by the release of NO by endothelial cells during the phase of hyperemia since it can be prevented by using N(G)-MonoMethyl-L-Arginine (L-NMMA), which is a selective inhibitor of eNOS. Importantly, NO dependence is influenced by location of the inflating cuff. Dilation upon hyperemia is completely blocked by infusion of L-NMMA when the cuff is placed distal to the measuring site, but only partially when the cuff is placed proximal to the echo probe [87]. The latter position of the probe creates an area of ischemia within the region of the probe, and therefore other vasoactive substances may play a role. Although in the past the predictive power of FMD was attributed to its NO dependency, a recent meta-analysis by Green et al. demonstrated that FMD measured after proximal occlusion is at least as predictive as measured after distal occlusion, although less NO dependent [88].

The assessment of FMD is still the most widely used method but it requires intensive training in order to achieve acceptable reproducibility. Besides technical factors, which are reviewed elsewhere, [86, 89], patient-related characteristics require a high level of standardization (Table 1). Measurements should be performed on the same time of day since FMD has a known diurnal variation [90]. A high fat meal [91] deteriorates endothelial function, and dietary components such as tea [92], Vitamin C [93], and chocolate [94] have short-term beneficial effects on endothelial function. Measurements should therefore always be performed in a fasting state before medication intake [95, 96]. Specifically for the pediatric setting, children frequently present with mild infections, which may impair endothelial function for up to 2 weeks [97]. Both mental stress [98] and room and skin temperature [99] influence endothelial function. It is therefore recommended to perform measurements in a dim-lighted and temperature-controlled (21–24°C) room and to carefully explain the procedure to studied subjects. Tobacco use should be avoided for 4 to 6 hours prior to measurements [100]. No guidelines are given regarding passive smoking, although this may be relevant for children, since childhood exposure to tobacco smoke leads to endothelial dysfunction in both children [101] and young adults [102]. Although we did not find evidence for a synergistic effect of smoking and childhood obesity on endothelial dysfunction, maternal smoking has been shown to correlate with several obesity-related risk factors in young children [103]. Gender matters in childhood since it influences endothelial function, with FMD being lower in boys [104]. Moreover, endothelial function differs during the menstrual cycle [105].

Contrary to adults, children present more variability in time to peak dilation in response to hyperemia. While in adults peak dilation usually occurs 60 seconds after cuff release, in children maximal dilation has been described between 40 and 120 seconds after-occlusion [106]. This effect is age dependent, and time to peak tends to drop with increasing age [104], advocating against a fixed moment in time to record maximal endothelium-dependent dilation. Effects of age on maximal dilation are conflicting with Donald et al. demonstrating no effect of age on FMD in a very large population of children (*n* = 7557) [99] and Sarkola et al. mentioning a decrease in FMD, which they explained by an increasing baseline internal diameter of the brachial artery [104].

Although we found 2 articles describing the absence of endothelial dysfunction in obese children [21, 107], numerous research groups have reported on impaired endothelial-dependent vasodilation, assessed with FMD, in this population [108–122].

### 3.2. Peripheral Arterial Tonometry

Peripheral arterial tonometry was developed as a novel technique to overcome the disadvantages of user dependence of FMD. The only commercially available and validated apparatus (Endo-PAT 2000) involves finger probes to measure arterial pulse wave amplitudes at the fingertip.

Guidelines for FMD measurements are also implemented for Endo-PAT measurements, although the interference of several factors was investigated separately. Research confirmed the effect of diurnal variation [123] and the effect of dietary components such as omega-3 fatty acids [27], polyphenol rich olive oil [124], chocolate [125], and tea [126]. Furthermore, measurements were influenced by drugs [81] and mental stress [127].

The Endo-PAT probes are placed on one fingertip of both hands and are inflated to produce a subdiastolic counter pressure, in order to provide fixation and prevent venous pooling. Pressure differences secondary to dilating arterioles in the fingers are measured. The procedure is initiated with a 5-minute baseline assessment; then a manometer cuff is inflated to supra-systolic pressures and the brachial artery of the nondominant arm is occluded. After 5 minutes, the cuff is released and reactive hyperemia is observed during 5 minutes. The software provided calculates a Reactive Hyperemia Index (RHI) and is defined as the ratio of the mean Pulse Wave Amplitude (PWA) between 90 and 150 seconds after deflation divided by a preocclusion period during 210 seconds before occlusion. This ratio is then divided by the same ratio for the control arm and multiplied by a baseline correction factor. A “Framingham reactive hyperemia” is also calculated. For this ratio, the period used to assess the hyperemia is between 90 and 120 seconds after occlusion since this period most strongly correlated with CV risk factors [128]. The ratio is log transformed as data indicate that values are not normally distributed.

Since the probes can never be placed proximal to the occlusion site, provided by the cuff, there is an effect of local ischemia. In addition, the hyperemic response that is measured is not entirely caused by endothelial NO production since it is not fully abolished by L-NAME [129]. Other mediators that contribute to vasodilation include prostacyclin (PGI_2_), a derivative of arachidonic acid that is secreted by endothelial cells. In normal arteries, NO has inhibitory effects on PGI_2_ release [130], and therefore the effect of PGI_2_ on endothelial dilation can vary in diseases associated with reduced NO bioavailability. A third mediator responsible for endothelial dependent dilation is Endothelium-Derived Hyperpolarizing Factor (EDHF), which has a larger influence on arterial tone in smaller vessels [131]. Fourthly, sympathetic tone can also modulate endothelial function, and an inverse correlation between sympathetic nerve activity and RHI has been demonstrated in healthy subjects [132].

Feasibility and reproducibility of Endo-PAT in adults are excellent [133]. The technique also appears highly reproducible in adolescents and causes hardly any discomfort [134]. To our knowledge, only 6 studies have been published in which endothelial function measured with Endo-PAT in obese children is described, and only 3 of them compared obese to lean children (Table 2). Results have been conflicting with Landgraf et al. [29] and Mahmud et al. [24] describing a lower RHI in obese children versus lean controls, whereas Tryggestad et al. [28] reported no difference.

Importantly, the Endo-PAT measures microvascular endothelial function, whereas FMD assesses endothelial function at the level of larger conduit arteries. Therefore, discrepant results are conceivable. Although Endo-PAT and FMD correlate well in healthy subjects [135], in patients with chest pain [136] and with coronary artery disease [137] such comparisons between the two techniques in both healthy and obese children are still missing.

Pubertal development and its associated hormonal changes complicate the study of endothelial function in children. RHI increases during puberty in both genders [138] and correlates to changes in estradiol and dehydroepiandrosterone sulfate in peripheral blood [139]. Both hormones upregulate eNOS concentration and activity [140].

Chen et al. demonstrated that age influences timing of the peak response of endothelial-dependent dilation measured with PAT [127] and noted shorter time to peak after 3-year followup, similar to FMD. These authors therefore propose to analyze the entire hyperemic response curve, instead of focusing on a very specific postocclusion time interval. By calculating the area under the curve, the problem of variability regarding time to peak dilation may be circumvented.

## 4. Cell-Based Methods for Evaluating Endothelial Dysfunction

The cell-derived methods mentioned hereafter are considered as biomarkers for endothelial status justifying their discussion in this review. Traditional CV risk factors influence their numbers and function. Fundamental and translational research have demonstrated that these biomarkers are more than plain bystanders whose numbers drop or rise as a reflection of endothelial homeostasis, but rather appear to be active players in the process of endothelial damage and repair.

### 4.1. Endothelial Progenitor Cells

In 1997, Asahara et al, in a murine model of hind limb ischemia, described for the first time that CD34 (a stem cell marker) positive mononuclear cells, which were isolated from human peripheral blood, could selectively incorporate into new capillaries in areas of ischemic injury resulting into neovascularization of the affected limb [141]. These cells were termed Endothelial Progenitor Cells (EPC). Since then, numerous experiments have investigated the biological characteristics of EPC. It has become clear that the original term EPC actually covers 3 different cell types, denoted as Endothelial Cell Colony Forming Unit (CFU-EC), Circulating Angiogenic Cells (CAC), and “true” EPC. In this paper, only the latter 2 will be given further consideration, because great controversies still exist on the origin, the proliferative potential, and the differentiation capacity of CFU-EC [142].

**Figure 2:**
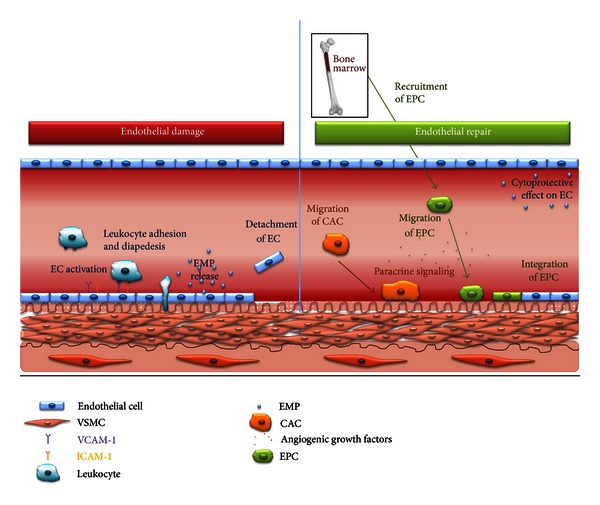
Mechanisms involved in endothelial damage and repair. Upon activation endothelial cells express adhesion molecules (i.e.; VCAM-1 and ICAM-1), which allow leukocytes to adhere, transmigrate, and initiate a cascade of inflammatory reactions and the release of EMP into the circulation. With significant endothelial damage, cells become senescent and are detached. This ultimately leads to the recruitment of CAC, monocyte-macrophage-derived cells that contribute to vascular repair by adhering to loci of endothelial damage, and producing angiogenic cytokines that induce the mobilization of EPC from the bone marrow. The angiogenic cytokines produced by CAC also serve as homing molecules with a chemotactic effect on EPC. As a consequence, EPC migrate to damaged endothelium and eventually integrate into the endothelial cell layer. Besides being released after endothelial activation, EMPs also contributes to endothelial homeostasis by cytoprotective effects on endothelial cells, including reduced apoptosis. EC, Endothelial Cell; VSMC, Vascular Smooth Muscle Cell; VCAM-1, Vascular Cell Adhesion Molecule 1; ICAM-1, InterCellular Adhesion Molecule 1; EMP, Endothelial MicroParticles; CAC, Circulating Angiogenic Cells; EPC, Endothelial Progenitor Cells.

True EPCs, alternatively called Endothelial Colony Forming Cells (ECFC), are released upon stimulation from bone marrow into the peripheral circulation and can incorporate into damaged endothelium (Figure 2) [143]. They are believed to be responsible for vasculogenesis and, as such, most closely fulfill the criteria for an Endothelial Progenitor Cell. EPCs appear in cultures of mononuclear cells after 7–21 days, have a cobblestone morphology, high proliferative capacity [144], and are able to form vessels *in vivo* [142]. Further analysis demonstrated that these cells express CD34 and Vascular Endothelial Growth Factor Receptor-2 (VEGFR-2) but not CD133 or CD45 (a pan-leukocytic marker) [145], even not at the mRNA level as was demonstrated by Case et al. [146].

The mechanism by which EPCs are released from the bone marrow is not fully understood but is the result of Matrix MetalloProteinase (MMP)-9 activation [147], in a NO-dependent mechanism [148]. As a consequence, reduced NO bioavailability not only leads to endothelial dysfunction, but also to the impaired recruitment of EPC to the loci of damaged endothelium as well, starting a vicious circle as described by Van Craenenbroeck and Conraads [149].

The technique most commonly used to quantify EPC is flow cytometry. Although this method is technically challenging due to very low numbers of circulating EPC, it is minimally invasive (blood sample via venipuncture) and therefore very convenient in children. Since there is no single, specific marker to identify EPC [150], a combination of markers is applied. Many different protocols have been developed between laboratories, with, however, poor intermethod agreement [151], which may have contributed to conflicting results in the literature. Schmidt-Lucke et al. introduced a modified International Society of Hematotherapy and Graft Engineering (ISHAGE) protocol for CD34+/KDR+ EPC enumeration gated on the basis of low SSC and low-to-bright CD45 fluorescence [152]. The authors concluded that it is in fact the CD45dim positive CD34+/KDR+ PC that correlate best with clinical characteristics of the studied patients with coronary artery disease. This protocol was presented to facilitate interlaboratory comparison and speed up EPC enumeration, yet technical recommendations for rare event analysis were not taken into account [153].

Since mature endothelial cells have limited regenerative capacity, EPC are necessary for endothelial repair. This notion is supported by a mathematical model of endothelial maintenance, which predicted a critical phase of endothelial cell defects that causes serious vascular damage. Such devastating consequences can be significantly delayed by incorporation of EPC [154]. Further *in vivo* evidence is provided by the fact that lower numbers of circulating EPC predict CV events and death in adults [155, 156] and by data showing that human EPCs are able to form fused vessels when implanted in immunodeficient mice [142].

Using flow cytometry it was demonstrated that EPC numbers correlate with endothelial function in CAD patients [157], in patients with type 1 diabetes [158], in young smokers [159], and in healthy subjects [160].

Müller-Ehmsen J et al. noted lower numbers of EPC in obese volunteers compared to healthy participants, while they observed a significant increase in EPC levels after weight loss [161]. Besides the direct effect of obesity, several other studies demonstrated reduced numbers of EPC in patients with obesity-related comorbidities such as hypertension [162, 163], hypercholesterolemia [164], and type II diabetes mellitus [165, 166].

Research in obese children has shown conflicting results. To our knowledge, there are only two papers comparing numbers of endothelial progenitor cells in obese children and lean controls. Unfortunately, each group used different protocols and different markers to detect these cells. Jung et al. were the first to investigate numbers of EPC in obese adolescents. They did not find significant differences in CD34+/KDR+/CD133+-cells, but did mention higher numbers of CD34-/KDR+CD133+-cells in overweight adolescents [167]. However, it has been shown that neither of these cells give rise to EPC in culture [146].

Arnold C et al. investigated whether numbers of circulating EPC related to physical fitness in obese children. Maximal oxygen uptake significantly correlated with CD34+ (*r* = 0.458) and CD133+/CD34+ cells (*r* = 0.456) in this population, yet the endothelial marker (KDR) was not assessed in this study [168].

In conclusion, studies comparing numbers of EPC between healthy and obese children that include CD45 as a marker and correlating their numbers to endothelial function in this specific population are not available and are eagerly awaited.

### 4.2. Circulating Angiogenic Cells

Circulating Angiogenic Cells (CAC) are grown from peripheral blood mononuclear cells after 4 to 7 days of culture in endothelial promoting media and fibronectin-coated plates. CACs have a low proliferative capacity and do not incorporate into endothelium, yet these cells restore damaged endothelium in a paracrine fashion by aiding in the recruitment and proliferation of, respectively, EPC and endothelial cells. CACs do not demonstrate an endothelial phenotype [169], but are hematopoietic cells, which closely resemble activated M2 monocytes [170]. Cell culture is the most widely used technique to determine their numbers and allows researchers to further explore their physiological activity. The migratory capacity towards Vascular Endothelial Growth Factor (VEGF) and Stromal cell-Derived Factor (SDF)-1*α* is commonly quantified using 7-day-old CAC cultures [171]. Both VEGF and SDF-1*α* recruit CAC to hypoxic tissues [172]. After an additional 3 days of culture, the supernatant can be collected and analyzed for protein secretion (such as VEGF) [173], and thus paracrine activity can be evaluated.

Numbers of CAC and their migratory capacity correlate with endothelial function [174]. Obese adults have lower numbers of CAC compared to healthy controls [175] and these cells display lower migratory capacity and reduced secretion of angiogenic growth factors, which can be reversed by weight loss [176]. However, results for obese children are still lacking.

NO is necessary for the migration of CAC towards VEGF [177], and therefore reduction in eNOS activity and reduced bioavailability of NO in obese patients could explain impaired migratory capacity of CAC in obese patients [178].

Leptin stimulates the migratory capacity of CAC, yet CACs of obese patients display a resistance against the leptin associated pro-angiogenic effect, which again is reversed by weight loss. Leptin resistance has been attributed to higher concentrations of Protein Tyrosine Phosphatase 1B (PTP-1B), a known inhibitor of the leptin signaling cascade. Higher levels of PTP-1B were seen in obese individuals, which returned to levels comparable to lean subjects after weight loss. Furthermore, blocking PTP-1B activity pharmacologically restored responsiveness to leptin in CAC of obese patients [176].

Adiponectin similarly enhances the migratory capacity of CAC towards SDF-1*α* [62]. This finding has been attributed to an upregulation of the receptor for SDF-1*α*, C-X-C Chemokine Receptor type 4 (CXCR-4) on CAC. Lower concentrations of adiponectin, seen in obese, could therefore further contribute to the functional deficit of CAC.

### 4.3. Endothelial MicroParticles

Endothelial MicroParticles (EMP) are small particles (100 nm to 1 *μ*m) shed from the plasma membrane by endothelial cells upon damage, activation, or apoptosis [179]. These microparticles are covered with surface antigens from the parental endothelial cell, making quantification as well as identification of the underlying process for their generation possible using specific flow cytometry markers.

Initially, EMP were considered as indicators of endothelial disruption [180], but their role has been redefined; instead of being mere markers, they also appear to elicit physiological effects. On the one hand, EMP themselves can further compromise endothelial function [181], whereas, on the other hand, they exert beneficial effects on endothelial cell survival and even promote endothelial regeneration [182].

Interestingly, EMP also act as vectors carrying RNA (including microRNA), DNA, and proteins to target cells [183]. Several studies have shown that the content of EMP depends on the trigger by which their parental cells were stimulated [184, 185].

The exact mechanisms that regulate the release of EMP *in vivo* are not completely understood, but involve an end in membrane phospholipid asymmetry and the expression of phosphatidylserine on the outside of the cell membrane, membrane budding, and eventually microparticle release.

EMPs are widely identified by their constitutive expression of CD31 (platelet endothelial cell adhesion molecule), but not CD42b. The absence of the CD42b (platelet-specific glycoprotein Ib marker) marker is used to avoid potential contamination with platelet microparticles that have the same size range as EMP. EMPs are to be detected in platelet poor plasma that was subjected to sequential centrifugation. In fact, careful attention has to be paid to all preanalytical and analytical steps, as variables in both phases may affect accurate microparticle enumeration and could be an important source of variability, interlaboratory discrepancies, and artifacts [186, 187].

Numbers of EMP are elevated in obese women and inversely correlate with endothelial function [188]. Van Ierssel et al. noted about 100 to 400 EMP per *μ*L blood in healthy volunteers [189], yet numbers may vary depending on methods used for sample preparation and analysis.

Data on EMP in children are scarce. Siklar et al. [190] provided indirect evidence that EMP numbers are higher in obese versus healthy children. By using a commercially available test (STA-PROCOAG-PPL Kit (Diagnostica Stago SAS, France)), they measured the procoagulant activity of phospholipids through assembly of the prothrombinase complex. Healthy children had a significantly longer microparticle release time, which was presumably due to a lower number of EMPs. Furthermore, Gündüz et al. showed that circulating vascular endothelial cadherin (CD144)+EMP are higher in obese and overweight children than in lean controls [191], but unfortunately, endothelial function was not assessed in this study. Further research is necessary to investigate the potential role of EMP as a biomarker for endothelial damage in obese children.

## 5. Pharmacological and Nonpharmacological Interventions to Restore Endothelial Damage

### 5.1. Weight Loss

The ultimate goal of therapy remains improvement of long-term physical health through permanent healthy lifestyle habits. Although weight maintenance is advised for younger children and a decline in BMI is achieved through growth, weight loss remains the cornerstone of therapy for obesity in adolescents [192].

Weight loss leads to an improvement of CV risk factors associated with endothelial dysfunction in childhood obesity [193, 194]. Because many studies have focused on the effect of multidisciplinary treatment approaches, little is known about the effect of weight loss alone on endothelial function in obese children. Kaufman et al. [195] studied the effect of a 5-month dietary intervention with a goal of a 5 to 8% decrease in total body mass in 15 overweight children. The study group consisted of six boys and nine girls (aged 11.4 ± 0.5 years). After the intervention, a significant decrease in weight, body fat percentage, and BMI was observed, with a trend towards improvement of endothelial function.

Woo et al. [196] compared the effects of weight loss alone to a combined treatment arm of diet and 6 weeks of exercise training in 82 overweight children, 9 to 12 years of age. In this study, an improvement of endothelial function was seen in both groups, albeit more pronounced in the diet plus exercise group (*P* = 0.01).

### 5.2. Exercise

Exercise training appears more effective than weight loss alone to improve endothelial function in obese children. As little as eight weeks of exercise training consisting of three 1-hour sessions of circuit training each week led to a significant improvement in endothelial function, even without weight loss in a randomized cross-over study involving 19 obese adolescents [197]. Two other studies, one involving endurance training and another applying aerobic interval training, confirmed the effect of exercise alone on endothelial function [198, 199].

In addition to the effect on factors leading to endothelial dysfunction [200], exercise training can more directly affect endothelial function through its effect on endothelial shear stress. Translational research using arterial biopsies of adults demonstrated that regular exercise leads to an upregulated expression of eNOS mRNA and higher eNOS protein and increased eNOS -phosphorylation levels in endothelial cells [201].

On top of increasing NO synthesis, exercise decreases production of ROS by reducing Nicotinamide Adenine Dinucleotide Phosphate (NAD(P)H) oxidase activity [202] and by enhancement of antioxidant capacity [203]. In addition, elevated shear stress, as seen with regular exercise training, can increase levels of BH_4_ [204] and thereby reduces eNOS uncoupling.

Exercise training has a significant effect on endothelial repair mechanisms, including both EPC and CAC. In adults with metabolic syndrome, 8 weeks of exercise training led to a significant increase in repair capacity of CAC [205]. These findings were confirmed by transplantation of human EPC into nude mice with defined carotid endothelial injury. In adults, a multitreatment approach including physical activity restored functional impairment of CAC [176]. In children, a program consisting of 12 weeks of exercise training increased the percentages of CD34+, CD133+ and CD34+/CD133+ cells and reduced carotid IMT [206]. In addition, 1 year of exercise training led to a significant rise in CD45low/CD34+/KDR+ EPC [207].

### 5.3. Pharmacological Interventions

Studies on the effect of pharmacological therapy in reversing endothelial dysfunction in children are very scarce. Therefore, potential candidates investigated in obese adults will be briefly discussed.

Since the withdrawal of both rimonabant and sibutramine, due the increased risk of psychiatric adverse events [208] and increased CV risk [209], respectively, orlistat a reversible blocker of lipase, is the only drug still available to aid weight loss. A recent meta-analysis of available data in children points out that the drug leads to 5 kg weight loss and 5 cm reduction in weight circumference after at least 6 months of therapy compared with placebo, but no improvement in the lipid profile nor insulin level was observed [210]. An open-label 3-month trial in adults consisting of a calorie-restricted diet and 120 mg of orlistat could not demonstrate a significant improvement in FMD [211].

Metformin is approved in many countries to treat insulin resistance in obese children. Although 3 months of metformin ingestion significantly improved both endothelial function and insulin resistance in adults with metabolic syndrome [212], adding metformin to a structured lifestyle intervention did not reverse insulin resistance in children [213].

### 5.4. Dietary Components

Dietary components have recently gained significant interest because of their potential benefit on endothelial function and presumed prevention of CV disease.

Adding vitamins C (500 mg/d) and E (400 IU/d) for 6 weeks to a program of diet and exercise further improved endothelial function in hyperlipidemic children [93]. Dangardt et al. were able to show that 3 months of omega-3 fatty acid supplementation improved vascular function in obese adolescents [27].

Although results from several randomized controlled trials with dietary components are quite promising, effects need to be confirmed in larger population-based studies.

### 5.5. Future Research Needs

As mentioned earlier in the paper, children are not just small adults and the speed by which physiological changes occur during puberty is tremendous. Puberty modulates endothelial function, and this improvement seems to be mediated through hormonal changes. The underlying mechanisms are not yet completely understood and deserve further investigation, as it may reveal key factors capable of ameliorating endothelial function later in life.

Further observation and perfectioning of flow cytometry and culture techniques have made EPC enumeration even more reliable. Yet studies on childhood and adolescent obesity that have adequately implemented these optimized strategies are lacking. In addition, the relationship of EPC and EMP with endothelial function also needs to be fully addressed in obese and overweight children.

And last but not least, whereas pharmacological interventions in obese children have been largely disappointing, inclusion of specific dietary components has shown to be highly effective in improving endothelial function in the short term. Further research, however, is needed to confirm whether these beneficial effects still remain at long-term followup.

## 6. Conclusions

Recent research has demonstrated the relevance of obesity-induced endothelial dysfunction, both in adults and in children. Fundamental and translational studies have led to significant understanding of cellular and molecular alterations held responsible for endothelial disruption. Unfortunately, research has until now mainly focused on the progression of atherosclerosis in obese adults, who have been exposed to the consequences of endothelial dysfunction during many years. Despite technical difficulties and ethical concerns, the investigation of endothelial function in obese children is a necessary step to further examine the initiation and progression of endothelial dysfunction, which is crucial to the development of treatment strategies.

Use of novel circulating markers can further unravel the delicate balance between endothelial damage and repair that causes endothelial dysfunction in obese subjects. 

Health care management should aim to avoid the catastrophic rise in CV morbidity and mortality, which will accompany obese children into adulthood. Therefore, multi-disciplinary prevention programs need to be set up and tested for their clinical effect. In order to speed up advances achieved in this domain, clinical research will largely depend on the effect of such interventions on the so-called surrogate endpoints, such as the correction of endothelial function.

## Figures and Tables

**Table 1:** Patient-related factors influencing clinical assessment of endothelial function in children.

Influencing factor	Solution
Time of day	Perform measurements between 8 and 12 am to rule out an effect of diurnal variation of endothelial function
Food	Perform measurement after an overnight fast
Medication	Ask patients to take their medication after the test
(Mild) infection	Postpone the test for >2 weeks
Active and passive smoking	Exclude smokers and/or record (parental) smoking habits
Gender	Check whether all groups are matched for sex
Age	Check whether all groups are matched for age and pubertal stage
Menstrual cycle	Girls in same phase of cycle or note the menstrual phase
Skin temperature	Allow sufficient patient acclimatization time; cover patients using a blanket
Mental stress	Avoid anxiety by providing patient information; perform the test in a quiet room

**Table 2:** Overview of studies using Endo-PAT to measure endothelial function in obese children.

Reference	Age (years)	Definition of obesity and overweight	Comorbidities	Parameter	Outcome
Mahmud et al. [24]	Obese: 13.4 ± 1.7; Lean: 14.0 ± 1.4	Obesity = BMI > 95th percentile	All insulin resistant, based on the HOMA score	RHI	Mean RHI was significantly lower in obese adolescents compared with controls (1.51 ± 0.4 versus 2.06 ± 0.4)

Metzig et al. [25]	12.4	Obesity = BMI > 95th percentile	15% with hypertension, 15% with dyslipidemia, 9% with OSAS, and 15% with impaired glucose tolerance	RHI	No significant effect of glucose ingestion on RHI

Kelly et al. [26]	12.7	Obesity = BMI > 95th percentile	?	RHI	No significant effect of exenatide therapy on RHI

Dangardt et al. [27]	15.7	?	?	Maximum dilation, area under the curve	Significant improvement of endothelial function after 3 months of omega-3 fatty acid supplementation

Tryggestad et al. [28]	Obese: 13.9 ± 2.5; Lean: 13.3 ± 3.0	Obesity = BMI > 95th percentile	?	RHI	No significant difference between obese and normal weight children

Landgraf et al. [29]	Obese: 11.8 ± 2.9; Lean: 12.9 ± 2.9	Obesity = BMI > 97th percentile; overweight = BMI > 90th percentile	?	RHI	Mean RHI was significantly lower in obese and overweight children compared with controls (1.28 ± 0.24 versus 1.96 ± 0.79)
